# How Much Did Urban Park Use Change under the COVID-19 Pandemic? A Comparative Study of Summertime Park Use in 2019 and 2020 in Edinburgh, Scotland

**DOI:** 10.3390/ijerph20217001

**Published:** 2023-10-31

**Authors:** Leyla Deniz Kiraz, Catharine Ward Thompson

**Affiliations:** Edinburgh School of Architecture and Landscape Architecture (ESALA), Edinburgh College of Art, The University of Edinburgh, Edinburgh EH3 9DF, UK; c.ward-thompson@ed.ac.uk

**Keywords:** urban parks, COVID-19, behaviour observation, park usage, lockdown, change over time

## Abstract

The importance of urban parks was highlighted during the COVID-19 pandemic, when a number of restrictions on social gatherings were in place and people’s movements were often restricted to their local neighbourhood. This study examined the changes in patterns of park use before and during COVID-19 to understand how the pandemic influenced such use. The methods involved behaviour observation and mapping, to offer a comparison of the use of parks in Edinburgh, Scotland, before and in the first year of the COVID-19 pandemic. The findings show an overall increase in use of urban parks during COVID-19, as expected, with significantly higher use in social areas, sports and fitness areas, and playground areas. However, while there was an overall increase in people visiting parks with others during COVID, in woodland areas there was an increase in lone visitors. This study shows the importance of parks for socialisation, exercise and children’s play, but also for spending time alone in natural areas during COVID-19. The value of urban parks at a time of social disruption, such as the pandemic, is highlighted, and their role in supporting a variety of urban dwellers’ needs points to priorities for future park planning, design and management.

## 1. Introduction

The importance of urban parks was highlighted during the COVID-19 pandemic, when a number of restrictions on people’s movement and on social gatherings were in place. In these circumstances, local parks assumed greater importance for many urban dwellers as a safe place that could legitimately be visited. While online surveys of self-reported visits to parks and other urban green or natural spaces during the COVID pandemic were undertaken by several researchers (e.g., Natural England [1], Olsen and Mitchell [2], and Stewart and Eccleston [3]), the opportunity to undertake objective studies of actual park use were limited. To the authors’ knowledge, there have been no studies reported to date that compare urban park use in the summer immediately preceding the pandemic (i.e., 2019) with urban park use in the same locations during the first summer under pandemic restrictions (i.e., 2020). It is this gap that this study addresses.

By utilising behaviour observation and mapping, this study gathered data on the use of urban parks before and during COVID-19. The first wave of behaviour observation was carried out before COVID-19, while the second wave was undertaken when the initial stages of the UK’s first national lockdown were lifted, although some COVID-19 measures were still in place (e.g., in relation to social distancing) [4]. The comparison of park usage and user profile before and during COVID-19 offers insights for planners, designers and policymakers in relation to the provision of urban parks that can serve urban citizens in times of crisis like the COVID-19 pandemic.

## 2. Background

### 2.1. The Place of Urban Parks

The increased number of people living in urban areas is associated with the transformation of urban environments and associated lifestyles, resulting in people spending most of their time indoors and being physically inactive, while most of their outdoor hours are spent in non-natural urban contexts [5]. Associated with sedentary and unhealthy modern lifestyles, and with a reduction in people’s opportunities for contact with nature [6], chronic physical and psychological diseases have increased dramatically [7]. With the growing understanding of the importance of salutogenic strategies to combat patterns of unhealthy lifestyles (e.g., Bai et al. [7]), there is an increasing interest in investigating how green spaces may affect and support human health and wellbeing (e.g., Ward Thompson [8]). The positive effects of nature on human health and wellbeing have been highlighted by many researchers [9,10,11,12], with urban public parks being a particular area of interest [13,14,15,16,17] because they are green spaces potentially freely available for all to use. An urban park can be described as a large area that is generally open and mostly green, designed for public usage and utilised for a variety of recreational and conservation purposes [18]. 

There is evidence that closer proximity to green areas for urban inhabitants and more green areas in urban settings could be associated with increased benefits for human health and wellbeing [19,20,21,22,23]. Moreover, engaging with nature in urban settings, e.g., via green areas, may have positive effects on physical and mental health [24,25], stress reduction [17,26,27] and life satisfaction [26], as well as offering a higher chance of social interaction [28,29]. The role of green areas within cities in maintaining and increasing wellbeing has attracted heightened attention as a result of COVID-19. According to a study on the UK population by Fujiwara et al. [30] (p. 9), COVID-19 caused a “decrease in life satisfaction, daily happiness and sense of purpose, and higher daily anxiety”, and was associated with the lowest wellbeing in all measures since the ONS (Office for National Statistics) began collecting national wellbeing data in 2011. The authors relate this decrease in wellbeing to a wide range of effects of COVID-19, together with the social distancing mandated by the government. During COVID-19, once severe restrictions on people’s movements were lifted, there is evidence that urban green areas helped to maintain the wellbeing of people while allowing social distancing [31]. Studies undertaken during the COVID-19 pandemic showed that interaction with urban natural environments helped people to relieve their stress [32,33] and contributed to enhancing their wellbeing levels [34,35]. For this reason, there is likely to be an increase in the demand for access to urban natural environments in times of crisis, such as in the COVID-19 pandemic [36]. Alongside that, however, among people who are at higher risk of the adverse effects of COVID-19, those who frequently visit green spaces may experience increased anxiety about maintaining social distancing, leading to worse psychological distress [33]. Therefore, people who experienced higher anxiety regarding becoming infected or who were at a higher risk of the adverse effects of COVID-19 may have been deterred from going to green spaces [37].

### 2.2. COVID-19 Restrictions on People’s Activities in Scotland

The first national lockdown in the UK in response to COVID-19 was in force on 24 March 2020 [38]. The lockdown rules included a ban on public gatherings of more than two people, the closure of non-essential shops, along with public places, communal parks and playgrounds. Leaving home was limited to essential purposes such as essential shopping, limited to once a day; taking exercise, limited to once a day and alone or with a household member but not in groups; for medical reasons or for caring for a vulnerable person; or for travelling to essential work that could not be carried out from home [39]. 

On 29 May, Scotland entered Phase 1 [4], in which, while people were encouraged to stay at home as much as possible, two households (up to eight people) were allowed to meet outdoors while maintaining social distancing rules, and people were advised to remain in their local area, travelling only short distances (a suggested maximum of 5 miles from home) to outdoor leisure and exercise [40].

Phase 2 was initiated from 19 June. Under this, people living alone or with children aged under 18 could form extended household groups whose members could meet indoors, but people were expected to continue staying in their local area, with travel distance limited to a maximum of around 5 miles for leisure and exercise. From 29 June, outdoor sports courts and playgrounds were allowed to re-open, as could retail outlets with street access and outdoor markets [41]. A further easing of restrictions was subsequently put in place. From 3 July, children aged 11 and under were allowed to play outdoors without social distancing, while children aged 12 to 17 were subject to physical distancing rules, and both age groups could meet with a maximum of two households (eight people) at one time. Moreover, the travel distance limit was relaxed [42,43]. 

Phase 3 was put in place from the 10 July. According to this, people from a maximum of three households (up to 8 people) could meet indoors, and a maximum of five households (up to 15 people) could meet outdoors. From 13 July, non-essential shops inside shopping centres could re-open, and organised outdoor play and contact sports could be pursued for children under 18; from 15 July indoor cafes and pubs, hairdressers, museums, cinemas and other visitor attractions and childcare facilities could re-open. From 22 July, other personal retail services could re-open, and, from 31 July, non-essential offices could re-open, and live events, indoor gyms and outdoor contact sports could resume [44].

### 2.3. Changes in Park Usage as a Result of COVID-19

In times of crisis, like the COVID-19 pandemic, people’s behaviours and routines are disrupted, even when formal restrictions are lifted, and the changes include their everyday routines regarding the use of urban green areas. With the lockdown limitations placed on indoor socializing and the closure of indoor leisure and sports facilities, for example, or because of the stress brought on by the pandemic, there was increased demand to use green areas by some. On the other hand, some people were deterred from going to outdoor areas, including green spaces, that might involve interaction with other people, because of concern about health (for them or a member of their household) and the risk of being severely affected by COVID-19 [37]. 

Several studies were conducted to understand the effect of COVID-19 on outdoor use in the UK. For example, Stewart and Eccleston [3] aimed to assess the impacts of the pandemic and social distancing on people’s outdoor visits and their engagement with nature. The first wave of their study (from 23 March to 28 May 2020) focused on the effects of the lockdown, while the second wave (mid-August to early September 2020) took a more comparative approach to understand the changes during lockdown and after the restrictions were relaxed [45]. Another survey that provided insight into the effects of COVID-19 on outdoor space use was ‘the People and Nature Survey for England’. It was developed based on the Monitor of Engagement with the Natural Environment (MENE) [46] and aimed to provide information on people’s interaction with natural environments during COVID-19 and its effects on wellbeing. Generally, surveys undertaken during the early months and years of the COVID-19 period confirm that there was an increase in the average use of green spaces, which was even higher after the lifting of the lockdown restrictions [1,2,3,45,47,48,49,50,51]. Moreover, many survey reports showed that people anticipated and/or experienced the benefits of green spaces for their mental health and wellbeing [1,47,48,49,50,51,52]. Women, younger people (aged 16–34), families, and those with good health reported spending more time outdoors, while older people (70+) and those with poor health reported spending less time outdoors during lockdown [3]. In association with this, average short walks, cycling and jogging levels also increased during the lockdown, reflecting the government restrictions on people’s movements, and people spent more time in local parks and woods and less time in coastal locations and remote rural areas [3]. However, after the most restrictive phases of the lockdown were lifted, visits to a variety of greenspaces, including remote areas, increased [45]. During and after the lockdown, the main motivation reported by survey participants was taking care of their physical and mental wellbeing [45]. Based on such surveys, around half of the population in Scotland expected to spend more time outdoors for leisure, recreation and exercise in the future [3,45].

Such studies evidence how the COVID-19 pandemic influenced attitudes to parks and green space usage and self-reported behaviour in visiting such areas. 

### 2.4. Understanding Human Behaviours in Urban Parks

Approaches to understanding park use include: observation studies [53,54,55,56], user and citizen surveys [57,58,59], interviews and mixed methods techniques [60]. 

According to Moore and Cosco [61], behaviour mapping can provide information on the environment–behaviour relationship. Behaviour observation and mapping is a structured technique which is commonly used to determine the usage of space. It is also a flexible technique that can accommodate the supply of various information needs. Ng [62] defined behaviour mapping as a record of how the behaviours of people are distributed in space, while Moore and Cosco [61] (p. 34) described it as “an unobtrusive, objective, observational method for measuring actual use of space” and set out detailed approaches to its application, drawing on earlier techniques, e.g., as described by Zeisel [63]. Compared to other techniques used to investigate park usage, behaviour observation and mapping have both advantages and disadvantages.

Park user surveys and interviews are valuable in helping researchers to understand people’s attitudes to particular places, their reasons for visiting and their experience of the place. Moreover, detailed personal information, such as socioeconomic status and physical and mental wellbeing status, can be gathered, as well as individual patterns of park usage over time not possible to achieve through behaviour observation. On the other hand, it is not always possible to reach the full range of park users in a survey, since the results would depend on who is able and willing to participate in the survey. Moreover, it is not possible to determine precisely the actual use of space at a specific time, since survey results would depend on who participated in the survey and their reported park usage routines. In comparison, behaviour observation and mapping techniques can provide accurate data on the actual use of a space. In addition, they can record behaviour that people do not want to report, that they forget or are unaware of, or that they do not find worth mentioning [62]. 

In the context of the COVID-19 pandemic, behaviour observation and mapping have been used in a few studies to record the behaviours of people in open spaces during the early years of the pandemic. These include Petrtýlová and Jaššo’s [64] investigation of a waterfront in Bratislava, Slovakia, and Rozman Cafuta’s [65] analysis of public open spaces in Maribor, Slovenia, in which behaviour observation and mapping were utilised to determine the list of activities. However, this is the first study the authors are aware of that provides accurate records of park usage during the early years of the pandemic and the behaviours and interactions between different park users which resulted, compared with park usage immediately pre-COVID-19. What is of particular interest goes beyond total park usage levels, in order to better understand the following: the different areas and types of park space that were most or least used, by what types of users; the observed behaviours and interactions between users during the pandemic; and, crucially, how this might have changed compared with pre-COVID-19 park usage. It is this gap in understanding that this study addresses, taking advantage of behaviour observations undertaken in urban parks before and in the first year of the COVID-19 pandemic in Scotland’s capital city, Edinburgh.

## 3. Methodology

### 3.1. Research Design

This study took advantage of an initial wave of data collection undertaken in urban parks in Edinburgh pre-COVID-19, in July 2019, to make a comparison with data collected in the same parks at the same time of year in 2020, during the first year of the COVID-19 pandemic but at a time when lockdown had been lifted, and restrictions on visiting parks (local or far away) were no longer in place. During this time, however, some restrictions (e.g., social distancing, the number of people who could meet up, closure of indoor gyms and restrictions on outdoor contact sports), as described in Section 2.2, were still in effect [44]. 

The initial survey selected ten urban parks, owned and managed by the City of Edinburgh Council, for behaviour observation (as part of a wider PhD study). The aim of the study was to investigate what effect the COVID-19 pandemic appeared to have on types and patterns of park use, compared with pre-pandemic data. This involved, firstly, identifying observation zones for each park according to their characteristics and ease of observation and, secondly, recording people’s use of, and behaviour in, these different zones. The changing patterns of use and behaviour between wave 1 and wave 2 of the survey were then compared.

The data collection and analysis involved five steps: ‘site selection’; ‘preparation for observations’; ‘on-site observation’; ‘post-processing and mapping’; and ‘analysis’.

### 3.2. Step 1: Site Selection

In Edinburgh, there is a long history of open spaces associated with the city structure, which has affected the character of the city [66]. There is a diverse range of parks of different size, amenities and settings, making them good experimental sites for this study, with a variety of park qualities that could attract a variety of users and accommodate a wide range of activities. This variety formed the basis of the initial choice of sites for the study.

**Figure 1:**
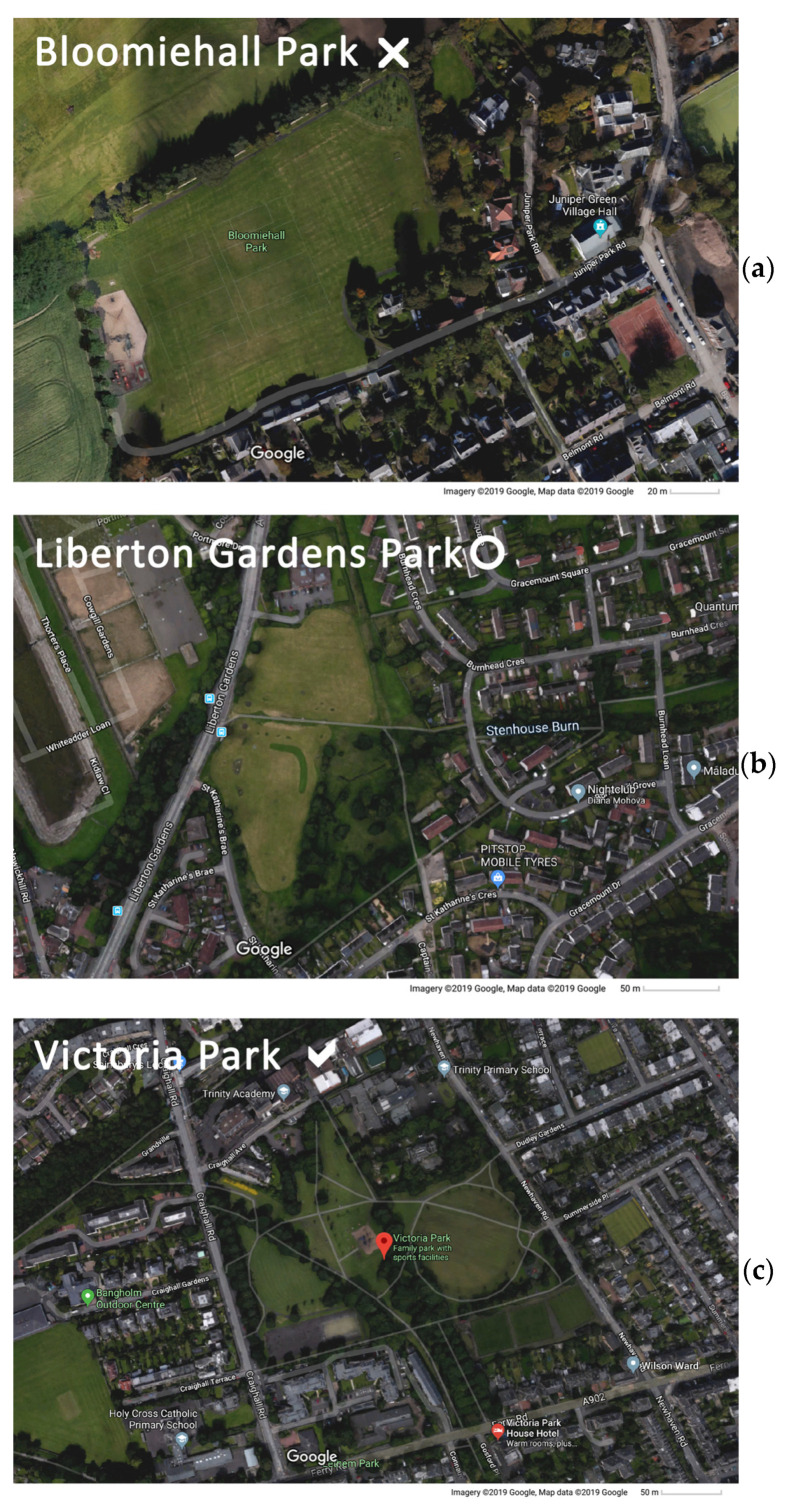
Illustration of selection of parks using Google Maps. Example parks in excluded, lower potential, and higher potential categories. (**a**) Bloomiehall Park, in the excluded category, satellite image [68]; (**b**) Liberton Gardens Park, in the lower potential category, satellite image [69]; (**c**) Victoria Park, in the higher potential category, satellite image [70].

**Figure 2:**
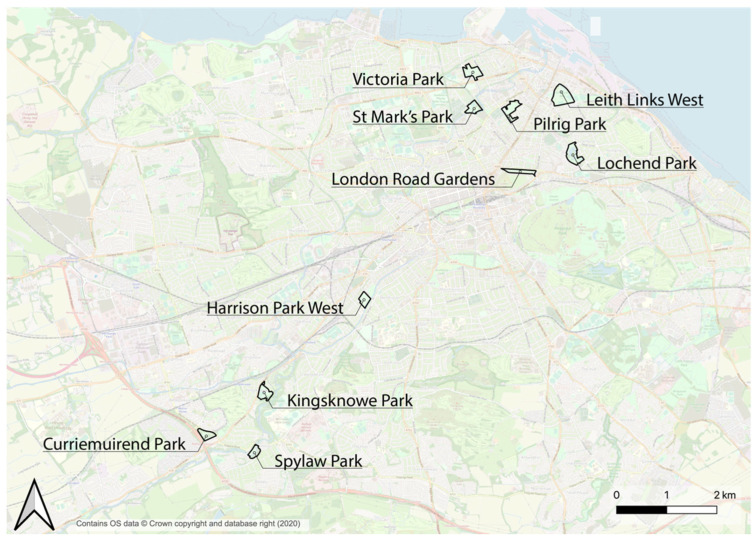
The 10 urban parks selected for behaviour observation and mapping study (map prepared by the authors which contains OS data © crown copyright and database rights, 2020).

In order to select the specific parks to be included in the study, the City of Edinburgh Council Atlas [67] was used. Several filtering operations were carried out using this tool to choose parks appropriate for behaviour observation and mapping studies. A size filter was applied to be sure that the parks were large enough to accommodate several activities while they could be observed by a single person, which would also provide comparability between the different parks in the study. Out of 1362 green spaces in Edinburgh, 38 were selected based on the following Atlas criteria: being within the public parks and gardens category (types of open space); having public access (access); being council-owned (ownership); being classified as a city park, community park or premier park (classification); and being between 2 and 10 ha in size (area). Later, out of the 38 parks meeting these criteria, 10 were selected for the study as having a higher potential to accommodate a wide range of qualities and activities, using Google Maps and aerial images to provide additional information (see Figure 1 for examples of parks excluded or included). The 10 parks chosen were as follows: St Mark’s Park, Pilrig Park, London Road Gardens, Victoria Park, Leith Links West, Lochend Park, Harrison Park West, Kingsknowe Park, Curriemuirend Park and Spylaw Park (Figure 2).

### 3.3. Step 2: Preparation for Observations

The second step was preparation for observations, which involved the creation of site maps [61,62,71], determination of observation zones [61], creation of the behaviour checklist and observation log [62,71] and scheduling observations. 

In order to produce the site maps to help in determining the observation zones for data entry, maps available on the Digi Maps platform were searched. Three options were considered for further investigation: OS Mastermap Green Space [72], Scotland Green Space [73] and Open Green Space [74]. Mastermap Green Space was chosen from among these as offering the most detailed and up-to-date maps. These were then transferred to QGIS to use as site maps. In producing site maps, the Open Street Maps within QGIS were used as a background for visualisation, to provide a sense of the site and surroundings.

**Figure A1:**
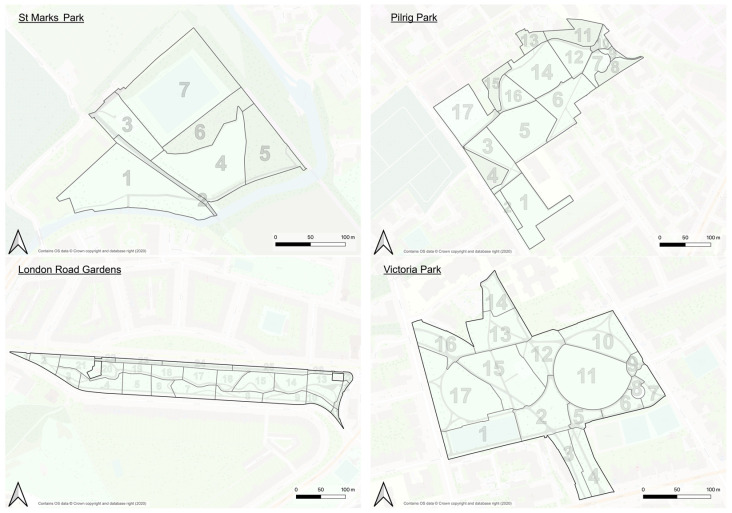

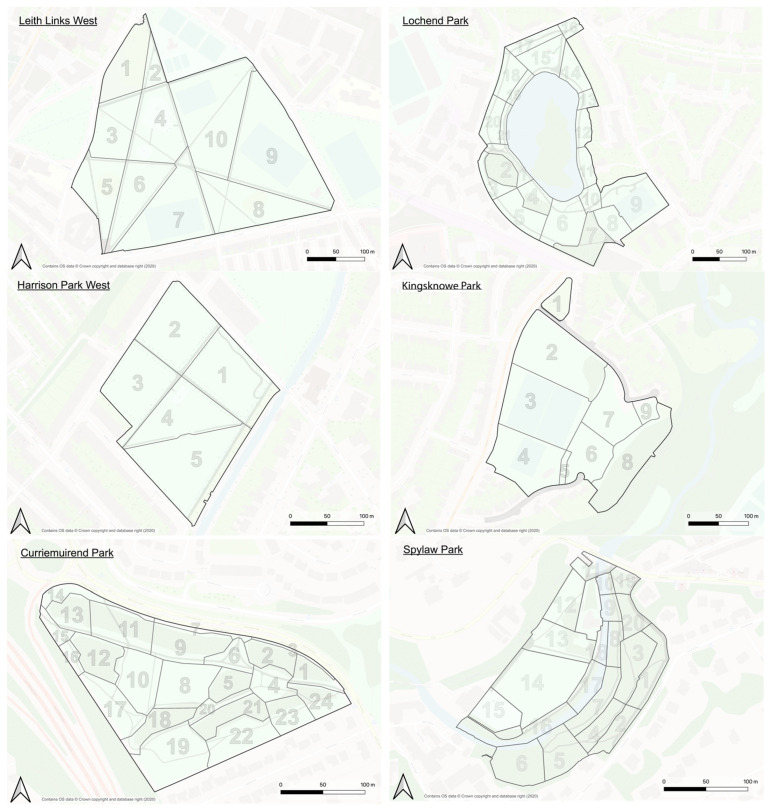
Maps prepared by the authors which contain OS DATA © Crown copyright and database rights, 2020. Numbers on the map indicate the location and sequence of observation zones.

The preparation step also involved dividing the site into observation zones, with the observation spot within each zone clearly identified. This provides coverage consistency across the whole site and between different observation rounds and observers (if more than one is involved). In this study, there was only one observer, so consistency in applying any protocol was ensured. In determining zones, the practicality of observation was the primary objective. Each zone could include several distinct site qualities or behaviour settings, so long as they could all easily be observed from a single location, taking into account any obstacles that could potentially block the view (trees, buildings, fences, topography, etc.). Although not a requirement, the observation spot was usually located near the centre of the observation zone. After deciding on observation zones and spots, the visit sequence was decided. This was based on the ease of travel between one zone and the next, with the ideal sequence ending up close to the start so that further rounds of observation could be undertaken, when needed, with minimum delay. At this point, the selected sites were investigated on the ground to finalise the observation zones and observation spots (see Table 1 and Figure A1). The size of the zones, and, consequently, the number of zones in each park, depended, in part, on the openness of the park. For instance, although they had similar park areas, there were only 5 observation zones in Harrison Park West because it had large open areas, while Curriemuirend Park had 24 observation zones since there are many trees on the site that obstruct the view. 

The user categories with their definitions and codes [62,71] were decided next, in order to create a checklist of the characteristics of site users to be noted and a log of their behaviours. The categories of user characteristics needed to be broad enough to be estimated consistently based on observation alone, while offering some insight into the variety of users observed.

In various research studies, activities in urban parks and green spaces have been investigated using diverse categories associated with the type of activities afforded [75,76,77,78], ranging from 3 broad categories (Adinolfi et al. [78]) to 17 detailed categories (Stigsdotter and Grahn [76]). A series of studies have also focused, more particularly, on the level of physical activity observed, determining the activity categories based on estimated energy expenditure. These are generally coded in three categories: sedentary, walking (or moderate) and vigorous activity [79,80,81,82,83,84], but sometimes in four categories: lying/sitting, standing, moderate activity and vigorous activity [85]. Such studies referred to the Compendium of Physical Activities, which was developed to be a classification of physical activities based on their energy expenditure, in order to provide comparability across studies [86,87,88] for Metabolic Equivalent of Task (MET) scores of activity levels (sedentary: 1.5, walking: 3 and vigorous: 6 [79]).

However, it is also not uncommon to broadly categorise park-based activities based on their likely intensity or energy-demand level in studies looking into activity types. The activity categorisation by Adinolfi et al. [78] (‘relaxation’, ‘sports’ and ‘walking’) and broad activity categorisation by Stigsdotter and Grahn [76] (‘Physically demanding activities’, ‘Exercise activities’, ‘Relaxing sporting activities’ and ‘Less demanding activities’) may be seen from this point of view, although Stigsdotter and Grahn’s category of ‘Socially demanding activities’ points to behaviours of interest where physical activity levels are not the defining characteristic. 

In this study, urban park activities identified from the literature were further considered for their validity and relevance, and the range of observed activities found in the Edinburgh parks were categorized according both to their physical activity and their social characteristics, to allow for both dimensions to be recorded for any one observed behaviour. The activity classes were further grouped based on their likely intensity. 

Age, gender, activity, social context (with whom, if anyone, the user interacted) and presence of groups were noted in the behaviour observation. 

A classification of activity and social categories was made, as shown in Table 2.

**Figure 3:**
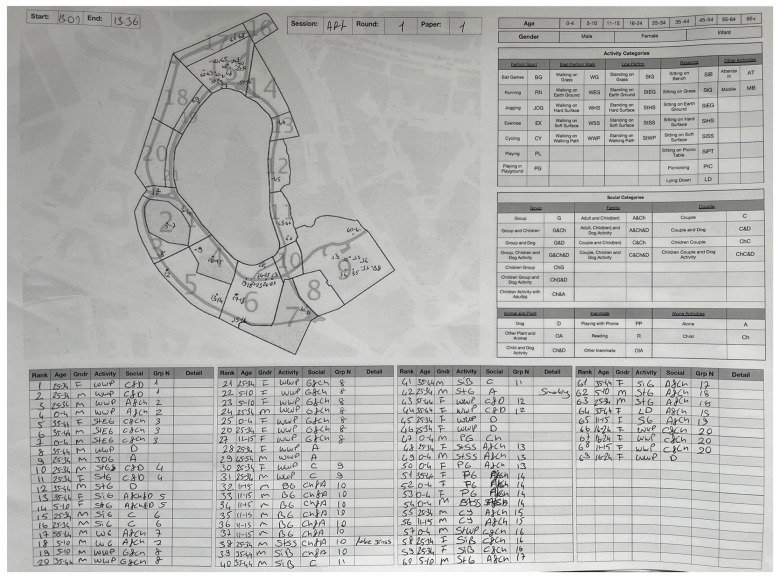
Example of completed Behaviour Observation log. Numbers on the map indicate the location and sequence of observation zones (observation log created and filled in by the authors, map prepared by the authors which contains OS data © crown copyright and database rights, 2020).

After deciding the categories, with the creation of a behaviour observation checklist and keymaps, observation logs were created, using paper and manual recording for ease in a park setting in all weathers (see Figure 3 for an example).

The ten urban parks selected for behaviour observation were visited between 8 July 2019 and 29 July 2019 in the first survey wave, and between 13 July 2020 and 31 July 2020 in the second survey wave. The observations were planned to begin on the second Monday of July and intended to be completed in two to three weeks, allowing some flexibility for schedule changes if the weather was particularly unfavourable. Three observation sessions were planned for each park: morning (between 9 and 10.30 am), afternoon (between 1 and 2.30 pm) and evening (5 to 6.30 pm) on the same day, and sessions were planned to cover weekdays only, to reflect typical patterns of use for most people’s normal working days. These observations were carried out as planned except for Victoria Park in 2019, when the evening session was postponed to later in July because of the heavy rain on the evening of the originally scheduled date.

### 3.4. Step 3: On-Site Observation 

In the behaviour observations process on site, each zone was observed in numerical order to ensure consistent sequencing of observations in different zones of each park, and scanned from each observation spot from left to right. In this way, the whole area was covered. This process was repeated three times for each observation session. Thus, a total of nine weekday observations were carried out for each park: three in the morning, three in the afternoon and three in the early evening. When an observation round took less time than anticipated (usually because of the lack of people on the site), more time was given between rounds to ensure the time for each of the three observation rounds was sufficiently spread out to be likely to capture the range of people visiting, regardless of how busy the site was. No unusual or planned special events were observed during the course of our observations.

Figure 3 illustrates a typical observation log.

### 3.5. Step 4: Post-Processing/Mapping

In this study, QGIS was used to digitise the data, and the EPSG: 27700 OSGB 1936 British Reference System was used as a spatial reference system. Each user characteristic noted in the behaviour observation was entered as a separate attribute. In this way, generating maps according to specific features (such as age, gender and activity) became possible. 

**Figure 4:**
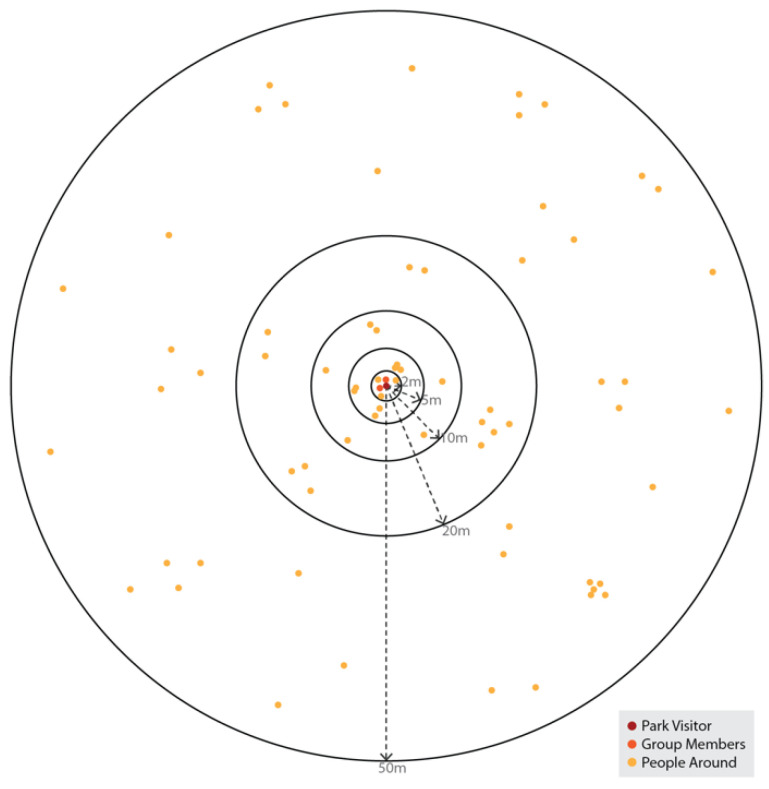
Calculation of experienced density of people (prepared by the authors).

In understanding people’s behaviour in parks, social proximity and interaction with others (whether planned or not) also plays an essential role, especially in the context of COVID-19 based rules on ‘social distancing’. In order to determine the possible interaction between people in the parks, data on social (or companion) group size (group size of individuals) and experienced density of people were produced. Group size is based on the number of people observed together or interacted with, regardless of whether they came to parks together or met at the park, while the experienced density of people was calculated based on the number of people around each person for different radiuses (2 m, 5 m, 10 m, 20 m, 50 m), including and excluding any group members (Figure 4).

**Figure 5:**
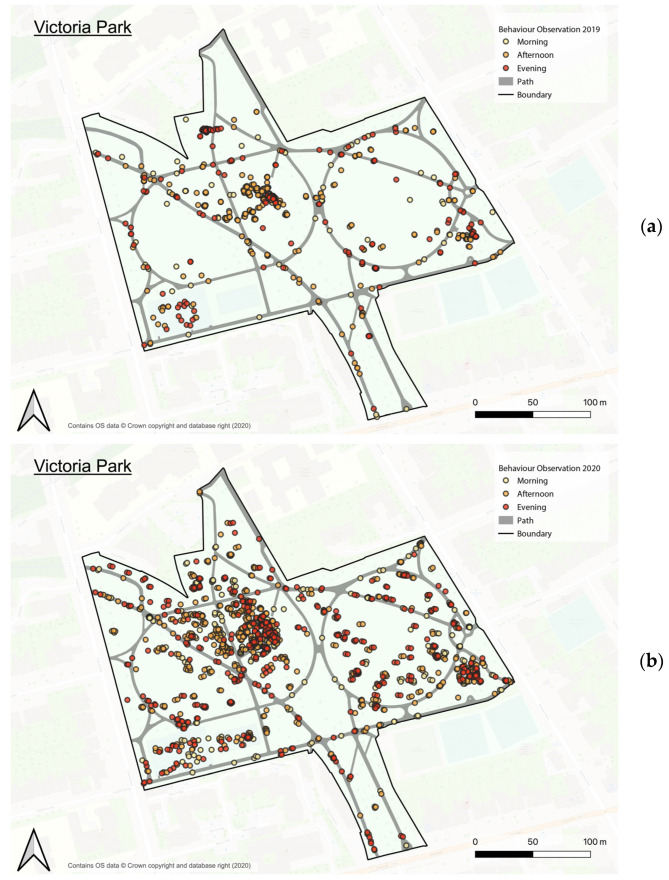
Example of behaviour maps based on observation sessions in Victoria Park, (**a**) in 2019 and (**b**) in 2020 (prepared by the authors, maps contain OS data © crown copyright and database rights, 2020).

**Figure A2:**
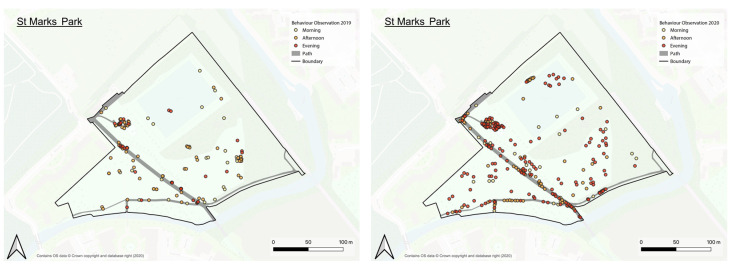

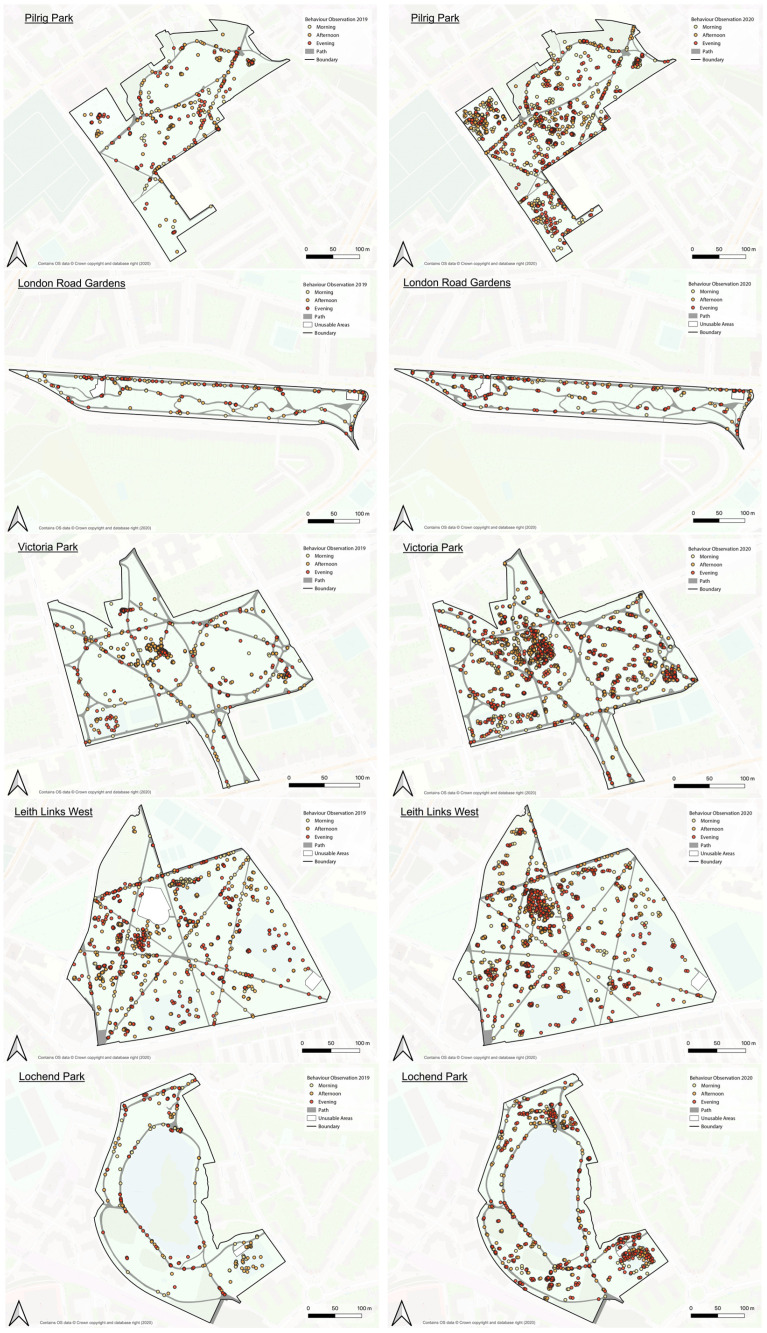

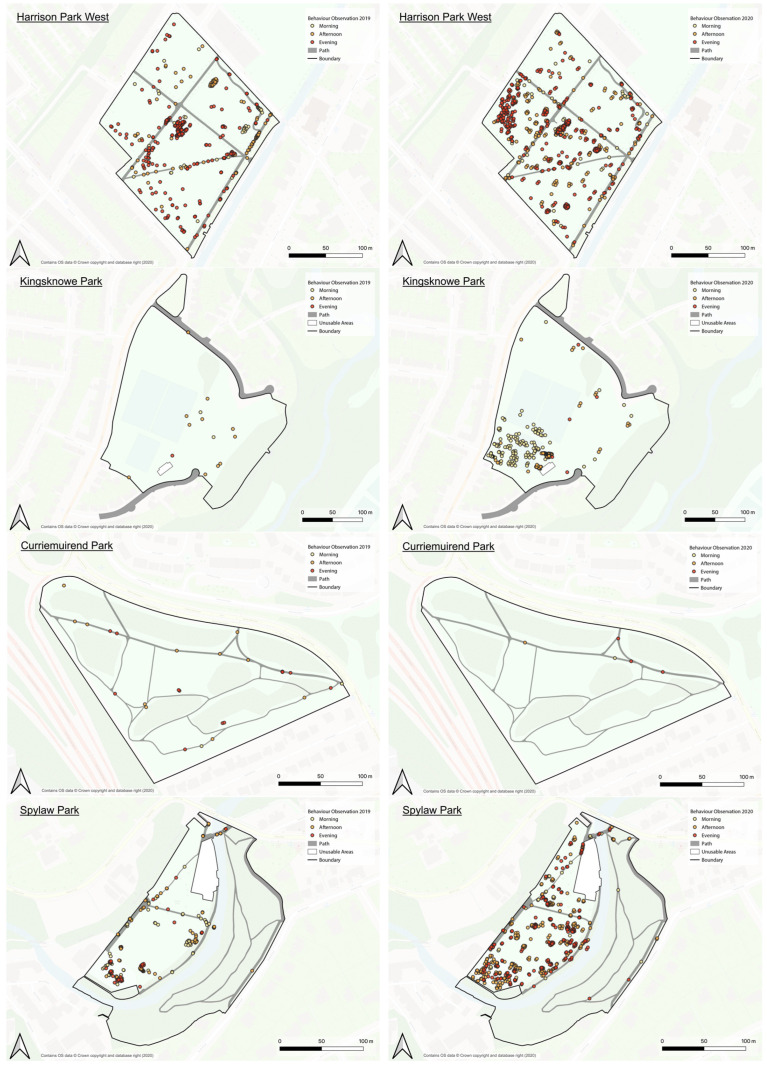
Behaviour maps based on sessions in each park. (**Left**): 2019; (**Right**): 2020. Maps prepared by the authors, maps contain OS data © crown copyright and database rights, 2020.

Behaviour maps were then generated based on the characteristics of users to understand site usage, as shown in Figure 5 (see Figure A2 for the full set of behaviour maps).

### 3.6. Step 5: Analysis

In this study, the resulting data were analysed to understand the park user characteristics, general park usage and changes between the two years of survey. In order to prepare the data for analysis, the users and accompanying characteristics were exported from QGIS (Version: 3.26.2-Buenos Aires, Open Source Geospatial Foundation (OSGeo)) to Microsoft^®^ Excel (Version: 16.78, Microsoft) as a CSV file to conduct a general overview. The data were later transferred to SPSS (SPSS Statistics 27, IBM) to conduct statistical analysis. To determine the change in park use and observed categories between 2019 and 2020, and differences among age groups and genders, chi-square tests were conducted. Moreover, for 2 × 2 contingency tables, odds ratios were calculated while, for larger tables, to determine the categories with the most significant change, adjusted residuals (z) were used (a threshold +/−1.96 was considered significant at the 95% confidence level). To determine the statistical significance of the change in group sizes, Mann–Whitney tests were used, and for experienced density of people, independent *t*-tests, were carried out. 

## 4. Results

There was an overall increase in park use. However, there was also a change in the pattern of use based on the proportion of users in different categories. While the overall proportion of male–female park users did not change significantly, all other patterns of park user category and behaviour type were significantly different between the two years—see Table 3. 

### 4.1. User Participation

**Figure 6:**
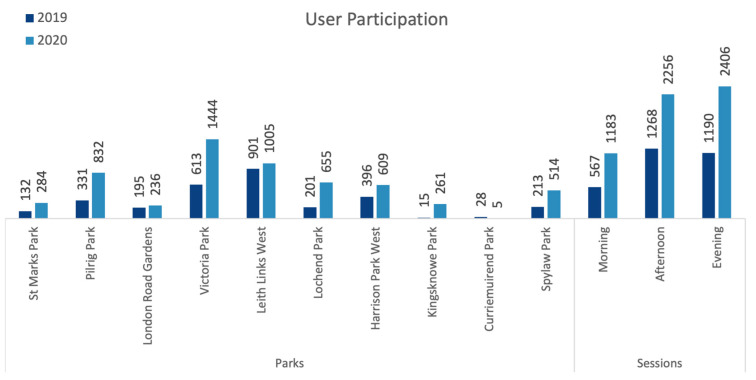
Comparison of total number of park users observed across all observation sessions in 2019 and 2020 for each park (data collected and graph prepared by the authors).

The overall number of people observed in parks in July 2020 was almost double that of the previous year, July 2019 (+93%) (see Table 3). Leith Links West is the largest of the parks studied and it was also one of the two most crowded parks in both years. However, the other most crowded park, Victoria Park, is the fourth largest park among the ten studied. There was an increase in 2020 (compared to the 2019 observation) in the number of people observed in nine out of ten parks. The parks with the most significant increase (Kingsknowe Park (z = 10.2) followed by Lochend Park (z = 6.9)) were the ones that were observed to host a lot of organised children’s sports activities. The most crowded session observed was on an afternoon in 2019, while it was on an evening in 2020. Notably, in both years, afternoon and evening sessions were more crowded than morning sessions (Figure 6). 

### 4.2. Age and Gender

**Figure 7:**
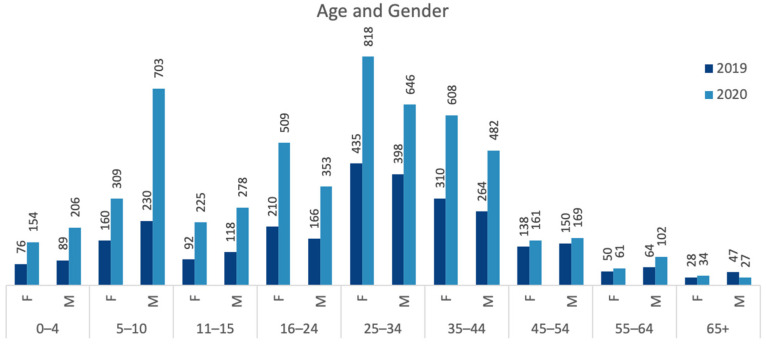
Comparison of 2019 and 2020 total park users observed, by age and gender (data collected and graph prepared by the authors).

The most numerous age group was 25–34, followed by the 35–44, 5–10 and 16–24 age groups, respectively, in both years. There was an increase in 2020 (compared to the 2019 observation) in all age groups except for the 65+ group. However, the decrease in the 65+ age group was because of the decrease in males in this age group (65+ males −43%, 65+ females +21%). The most significant rise was in the 5–10 age group (+159%) (z = 5.4). Notably, the number of 5–10-year-old boys tripled. The increase in the 5–10 age group was followed by numbers in the age groups 11–15 (+140%), 16–24 (+129%) and 0–4 (+118%). Overall, the number of males was significantly higher in children (aged 0–15) (z = 13.3), while females were higher in younger adults (aged 16–24) (z = 6.6) and young to middle-aged adults (aged 25–44) (z = 9.2) (χ2(4) = 214.896, *p* < 0.05) (see Figure 7). 

### 4.3. Activity Categories

**Figure 8:**
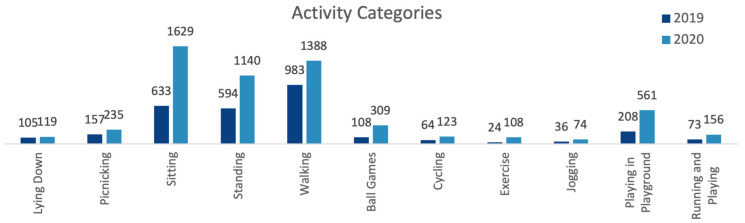
Comparison of 2019 and 2020 total park users observed, by activity categories (data collected and graph prepared by the authors).

The main activity category observed was ‘walking’, followed by ‘sitting’ and ‘standing’ in 2019, and ‘sitting’, followed by ‘walking’ and ‘standing’ in 2020. There was an increase in 2020 (compared to the 2019 observation) in all activity categories. The most dramatic and statistically significant increases were in ‘exercise’ (+350%) (z = 3.8), ‘ball games’ (+186%) (z = 3.5), ‘playing in playground’ (+170%) (z = 4.2) and ‘sitting’ (+157%) (z = 6.8) (see Figure 8).

**Figure 9:**
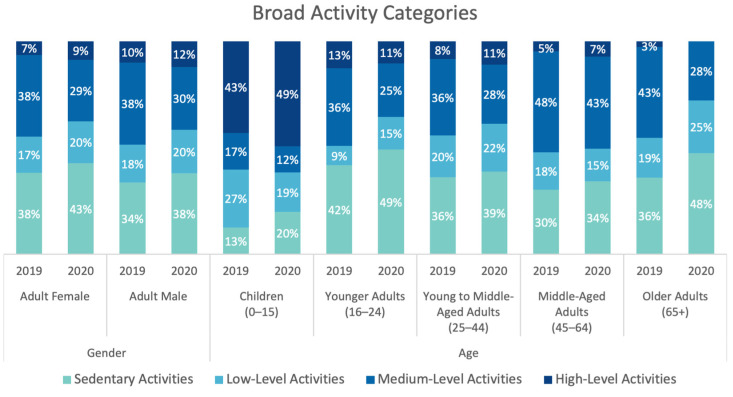
Comparison of 2019 and 2020 distribution of broad activity categories, by age and gender (data collected and graph prepared by the authors).

Overall, the distribution of broad activity categories in adult visitors was statistically significant between genders (χ^2^(3) = 27.920, *p* < 0.05). ‘High-level activities’ were more frequently undertaken by males (z = 4.5) while ‘sedentary activities’ were more frequently undertaken by females (z = 3.9). When age groups were investigated, it was found that children (below age 16) mostly undertook ‘high level activities’ (z = 40.2), younger adults (16–24) ‘sedentary activities’ (z = 11.5), young to middle-aged adults (aged 24–44) ‘sedentary’ and ‘medium-level activities’ (z = 9.4 and z = 7.3, respectively), and middle-aged (45–64) and older adults (65+) ‘medium-level activities’ (z = 13 and z = 2.4, respectively) (χ^2^ (12) = 1925.930, *p* < 0.05) (see Figure 9). 

### 4.4. Social Categories

**Figure 10:**
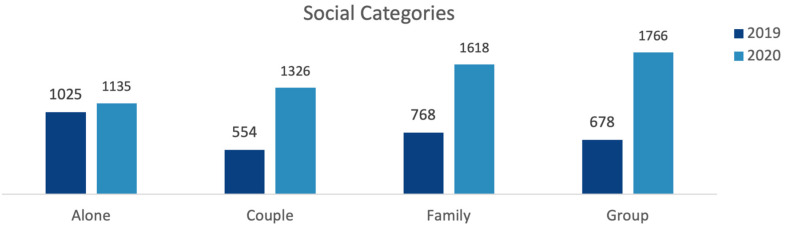
Comparison of 2019 and 2020 total park users observed, by social categories (data collected and graph prepared by the authors).

The top social category was people who were ‘alone’, followed by ‘family’, in 2019, while it was a ‘group’, followed by ‘family’, in 2020. There was an increase in 2020 (compared to the 2019 observation) in the number of people in all social categories, with ‘group’ activities having the most significant increase (+160%) (z = 7.8). However, the proportion of ‘alone activities’, as a percentage of activities undertaken within all social categories, significantly dropped (from 33.9% to 19.4%, z = −15) (see Table 3 and Figure 10). 

**Figure 11:**
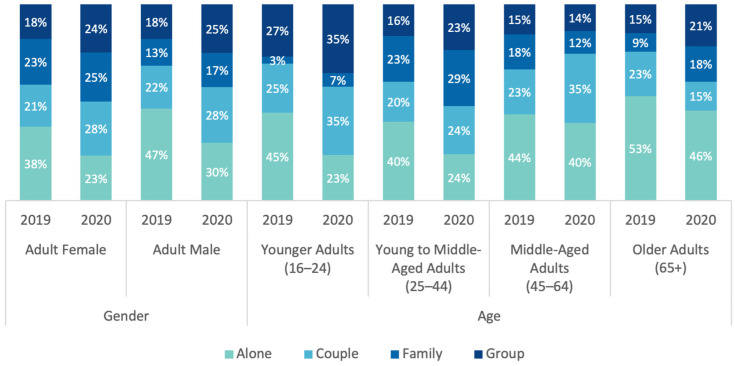
Comparison of 2019 and 2020 distribution of social categories in adult visitors, by age and gender (data collected and graph prepared by the authors).

When the distribution of social categories by gender and age groups in adult visitors was investigated, it was seen that adult visitors came to parks mostly alone, regardless of gender and age group, in 2019. However, in 2020, the percentage of lone visitors decreased in all age and gender groups. In 2020, the most frequently observed social category was ‘couple’ and ‘group’ in younger adults (16–24), ‘family’ in young to middle-aged (25–44), and ‘alone’ in middle-aged (35–64) and older adults (65+). Overall, a significantly higher percentage of males (z = 6.9) came to parks ‘alone’ than females, while a higher percentage of females (z = 8.3) came as a ‘family’ than males (χ^2^(3) = 86.613, *p* < 0.05) (see Figure 11). In summary, proportionately more adults visited a park with company, rather than alone, in 2020 compared to 2019. 

When the presence of dogs associated with visitors was investigated, although the number of people with dogs increased, the percentage of people with dogs in relation to overall visitor numbers decreased (the odds of visitors with dogs in 2019 were 1.63 times higher than in 2020).

### 4.5. Group Size and Experienced Density of People

**Figure 12:**
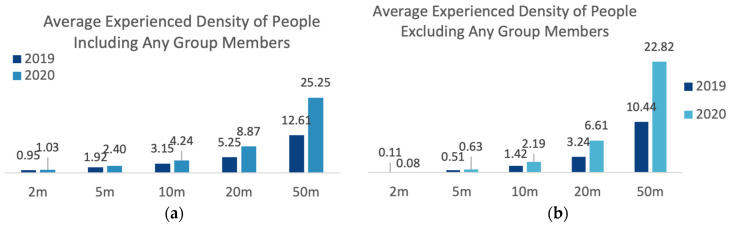
Comparison of 2019 and 2020 average experienced density of people. (**a**) Including any group members; (**b**) Excluding any group members (data collected and graph prepared by the authors).

The average group size in the 2019 observation was 3.17 and it was 3.45 in 2020, which also confirms that users came to parks in larger groups in 2020 compared to the previous year. The average experienced density of people, including any group members, within 2 m distance increased by 8% in 2020 compared to the previous year, while when excluding any group members, the average experienced density of people within 2 m distance decreased by 26%. At larger distances (5 m, 10 m, 20 m, 50 m), the average experienced density of people increased between 2019 and 2020, including and excluding any group members (see Figure 12). Considering the overall increase in the number of people visiting parks in 2020, and the fact that more people preferred to come to the parks with company, it is evident that people preferred to stay away from other people (who were not their companions) within the short distance of 2 m. 

### 4.6. Park Usage

**Figure 13:**
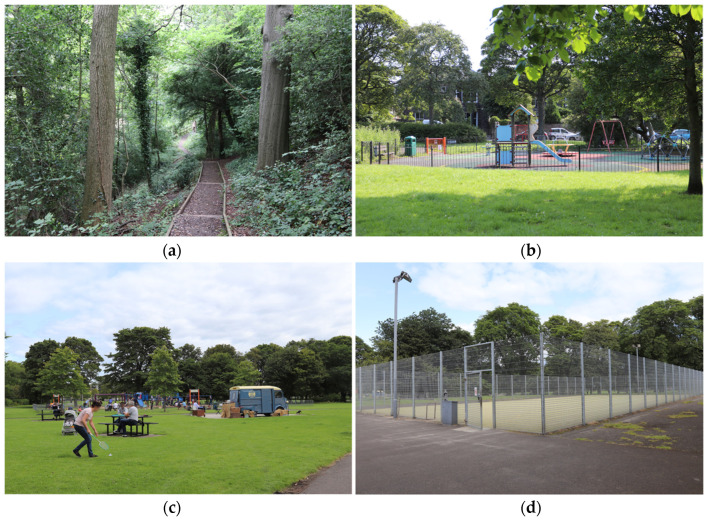
Images from parks to illustrate types of area: (**a**) Woodland; (**b**) Playground; (**c**) Social area; (**d**) Sports (photographs taken by the authors).

The total number of people observed increased by 93% in 2020 compared to the previous year; however, the changes varied in specific types of park areas (see Figure 13). The number of people in playgrounds increased by 117%, in woodlands by 168%, in sports and fitness areas by 276% and in social areas by 315%. The increase in social (z = 7.6), in sports and fitness areas (z = 5.5) and in playground (z = 2.2) was significant. On the other hand, the number of people observed on paths rose by only 28%, while the percentage of people observed on paths in relation to overall visitor numbers significantly decreased (z = −11.3). 

Overall, there was a significant difference in social categories in different park areas (χ^2^ (15) = 1508.503, *p* < 0.05) between 2019 and 2020. ‘Family’ and ‘group activities’ in social areas (z = 4.8 and z = 3.9, respectively), ‘family activities’ in playgrounds (z = 23.1), ‘group activities’ in sports and fitness areas (z = 8.2) and ‘alone’ and ‘couple activities’ in woodlands (z = 4.3 and z = 6.3, respectively) were significantly higher among all social categories in 2020. In 2019, only ‘alone’ and ‘couple’ activities were observed in woodlands. However, in 2020, although ‘family’ and ‘group’ activities were also observed in woodlands, the percentage of ‘alone’ activities there increased among all social categories in contrast to the overall decrease in the percentage of ‘alone’ activities. Moreover, the odds of lone visitors in woodlands—compared with the rest of the park areas—in 2020 were 3.17 times higher than in 2019 (χ^2^ (1) = 10.177, *p* < 0.05). In sports and fitness areas, the highest observed social category was ‘group’ in both years, and there was an increase in ‘group’ activities compared to other social categories. In social areas, there was an increase in the number of people in all social categories; however, the percentage of ‘alone’ activities reduced, ‘couple’ and ‘family’ activities roughly remained the same and ‘group’ activities increased. In playgrounds, the highest percentage of the social category was ‘family’ in both years; nonetheless, the highest percentage of increase was seen in ‘group’ activities. 

When the change between two years in adult visitors was investigated, it was seen that the highest increase was younger adults (16–24) in social, sports and fitness, and playground areas, while it was young to middle-aged adults (25–44) in woodlands. Notably, the number of older people (65+) decreased in playgrounds and woodlands. However, while no older people (65+) were observed in social areas in 2019, there were some older people observed in these areas in 2020. 

The most crowded area in terms of the experienced density of people, including any group members, was social within a 2 m radius (1.4 people on average), while it was playground in 5 and 10 m radiuses (2.9 and 6.6 people on average, respectively). On the other hand, the least crowded area was sports and fitness areas within a 2 m radius (0.4 people) and woodlands within 5 and 10 m radiuses (0.8 and 1.3 people on average, respectively). Excluding any group members, the most crowded type of area within 2 m, 5 m and 10 m radiuses was playground (0.2, 1.6 and 5.1 people on average, respectively), while the least crowded type of area was woodlands (0.0, 0.1 and 0.5 people on average, respectively).

## 5. Discussion

The number of people observed in 2020 was almost double the previous year. This increase would likely be related to the effects of COVID-19 on people, given previous restrictions on indoor gatherings and ongoing concerns about the dangers of infection when gathering with others indoors. Urban parks were some of the few suitable places to enjoy fresh air, socialise and exercise during this period. When the use of different types of areas in parks was investigated, it was seen that some areas, like social areas, sports and fitness areas and playground areas, had a higher proportional increase in user participation than the rest of the park areas. Interestingly, the percentage of people observed on paths had a smaller increase compared to overall park users. This suggests that the increase in the number of people mainly resulted in people visiting the park as a destination, rather than merely passing through it.

During the second survey wave, people could meet outdoors in groups of a higher number of people, and from more different households, compared with indoors [44], and there was general encouragement to meet outdoors instead of indoors. Beyond formal government restrictions and advice, people were themselves aware that meeting outdoors could be safer than meeting indoors. This no doubt led more people to meet outdoors and use urban parks for socialising, and explains the increase in the use of social areas in parks and the increase in average group size. Along with the increase in the number of park users, the number of lone people observed within a 2 m radius of others decreased. This indicates that people were trying to socialise outdoors while respecting government advice on social distancing in the 2020 observation.

Beyond this, there was a dramatic increase in exercise and ball games observed. This may have been related to the COVID-19 closure of gyms and indoor sports halls, as well as people’s preference for doing such activities outdoors. From the 13 July 2020, organised outdoor play and contact sports were allowed for children under 18 [44]. Reflecting that, there was an increase observed in organised children’s groups playing ball games in 2020 as well as a high increase in the number of people across all ages observed in sports and exercise areas, which shows the importance of the use of parks when indoor sports halls and fitness centres were closed. 

In both years, female adults were involved in a higher percentage of ‘family activities’, although the share of ‘family activities’ within all social categories increased in both men and women in 2020 and the gap between genders decreased. One of the effects of COVID-19 for many people was an increase in time spent on childcare hours, with a decreased gap in gendered responsibilities for this [89], which could be one of the reasons for increased family activities observed in parks. COVID-19 initially resulted in a loss of play and peer interaction for children, which was still a problem in the summer of 2020 [90]. There was a dramatic increase in numbers ‘playing in a playground’ in 2020, compared to the previous year. Playgrounds in parks are suitable places for children to play and for peer interaction, and the increased number of those observed ‘playing in a playground’ in 2020 shows the importance of these areas for people during COVID-19. 

Different from the rest of the park areas, there was an increase in the share of lone activities in woodlands. During COVID-19, people had lower wellbeing and higher anxiety levels [30], and interaction with nature could help them to be relieved from stress [32] and increase their wellbeing levels [34,35]. In hard situations, people may find it difficult to conduct complex interpersonal relationships, and thus may seek to engage in simpler connections, such as those formed with plants, animals or even inanimate objects (Searles, 1960, cited in reference [76]). Woodlands were found to be generally less crowded places compared to the average across all types of park areas and, for this reason, they appeared to be suitable places in urban parks for people who preferred to be alone and avoid social contact. 

There was also a change in the timings of the park use. Unlike in 2019, in 2020, the most popular time to visit was the evening. This difference could be related to the change in people’s routines because of the COVID-19 pandemic. For instance, during the observation in 2020, some people were working from home, and the evening could be a suitable time for them to visit parks if they had been indoors and at home all day. 

Many older people, being at higher risk of COVID-19 and its effects, developed a high level of anxiety, and even though the COVID-19 restrictions were eased during the summer of 2020, many were still afraid to go outside [91]. Furthermore, older adults (65+) had a higher likelihood of reporting concerns about their health when going outdoors and about them (or a member of their household) being at risk of being severely affected by COVID-19 as a barrier to visiting green spaces [37]. This could explain the overall decrease in the 65+ age group, while the number of people observed in parks in all other age groups increased in 2020 compared to 2019. This is in accordace with the results of the YouGov survey conducted in the UK during the pandemic, which found that older people (65+) were less inclined to visit green spaces compared to adults of younger age [37]. However, when age and gender categories were investigated, it was seen that the decrease in those aged 65+ was because of the decrease in males in this age group. This could be related to the fact that a higher percentage of males in this age group were shielding (among people who were 55 years old and older, 9.14% of females and 12.32% of males were shielding in the UK on July 2020 [92]), although it is not enough alone to explain the difference. Other possible explanations could be men having higher infection rates in older generations [93], higher severity (higher rates of hospitalisation [93,94] and admission to the intensive care unit [93,95]), and higher mortality rates [93,96,97,98]. Along with that, women claimed to be taking COVID-19 more seriously, and had a higher rate of following rules than men [99] and of taking protective measures, including choosing outdoor socialisation instead of indoors [100]. Another factor that could be related is that, although men were more vulnerable to COVID-19 physically, women were more affected psychologically [97,100,101]. For this reason, women with higher anxiety might need green areas to be relieved from stress. However, the reasons behind the difference in genders in this age group cannot be explained from our data alone and are worth investigating further.

This study shows that there were many changes observed in park usage and user profile associated with COVID-19 in 2020, compared to 2019. What is less clear is whether the changes that happened during COVID-19 will eventually be reversed, or whether newly established patterns of park use will persist. For instance, a lot of people started to use fitness apps during this time [102] and became used to outdoor exercise. It would be valuable to explore whether those people continue to visit parks for fitness purposes even many years after COVID-19 first spread. 

### Limitations

In this study, the categories were broad enough to minimise problems arising from inaccuracies in categorisations by the observer, e.g., in estimating age or levels of physical activity (the ‘observer factor’), while making them detailed enough to provide useful information for understanding changes in patterns of park use. However, it should be noted that the determination of age groups and genders relied on the observer’s judgement based on the physical appearances of the people observed. For this reason, age groups, for instance, can only be considered approximations. In future studies, especially if several observers are used, test-retest and inter-rater reliability tests could be carried out to help refine the category classes and ensure their robustness. Moreover, this study only investigated the characteristics and behaviour of park users during a limited period of the year, and only on weekdays, which means the findings may not include some behaviours and/or user types. Furthermore, this study only investigated selected urban parks in Edinburgh and their users at particular times on weekdays. While a strength of the study is its comparison of observations in the same locations, using the same protocols and at the same time of year over two, successive years, before and during the COVID pandemic, the user groups and activities of the chosen urban parks may differ from other parks in Edinburgh or other cities and different times of year. In addition, while comments on the findings are informed by other recent studies which involved surveys of people’s self-reported attitudes and behaviour, our data were reliant on observation alone and so can only report on the patterns of park use, not the reasons behind those patterns.

## 6. Conclusions

This study contributes to knowledge on the effects of COVID-19 on the use of urban green spaces. Urbanisation and modern lifestyles have resulted in decreased contact with nature which can impact people’s ability to maintain health and wellbeing, as supported by many research studies. Urban expansion and reduced access to open and natural spaces associated with people’s residences underlines the need for urban parks, which can provide a place for fresh air, physical activity, socialisation and recreation. Urban parks’ role in maintaining and increasing human health and wellbeing, especially in times of societal crisis, have gained greater recognition as a result of COVID-19. 

It is important to understand current park usage in order to provide urban parks that serve people’s needs, including making necessary upgrades to existing ones. Behaviour observation and mapping is a valuable method to understand and investigate park usage as it can provide accurate time- and location-based data on park users, which can be used in comparison studies focusing on changes happening over time, after interventions or between different sites. This study used such a method to compare the patterns of park usage prior to and during the COVID-19 pandemic. 

According to the comparative analysis of ten urban parks in Edinburgh before and during COVID-19, the number of park users almost doubled during COVID-19, with a significant rise observed in children aged 5–10 and a higher user participation observed in areas like social areas, sports and fitness areas and playground areas. In addition, the proportion of visitors who came as a group increased significantly, which, together with the significant increase in the use of social areas, shows the importance of parks for socialisation when indoor socialisation was limited. Moreover, there was a significant increase in exercise and ball games, as well as the use of sports and fitness areas, highlighting the importance of parks when indoor sports and fitness facilities were closed. There was also a significant increase in visitors to playgrounds in parks, which shows the importance of parks for children’s play during COVID-19. Another notable finding is the increase in people observed alone in woodlands, despite the overall reduction in the proportion of people visiting parks alone in 2020. This finding, combined with the lower experienced density of people in woodlands, highlights the importance of parks for people that are seeking to spend time alone in nature, recognising this as a contrast to those wanting to socialise or be with other people. In summary, there was an increased use of urban parks for various purposes like socialisation, exercise, play and spending time alone in nature during the COVID-19 pandemic. The gendered difference in park use by over-65-year-olds also points to issues of potential concern in understanding how the outdoor environment can support older people’s health, and particularly, perhaps, older women’s health, in times of crisis such as the pandemic. 

This study provides insights into the use of parks in Edinburgh in times of crisis. The comparative analysis of the use of parks before and during COVID-19 offers a basis for future studies on understanding the effect of the pandemic on users and their behaviours in parks. A repeat of the behaviour observation studies in future years would provide an opportunity to determine the change in the use of parks over a longer time post the introduction of COVID-19. This type of study could also be repeated in other cities to develop a comparative basis for understanding differences and changes in park usage over time in varying urban contexts.

## Figures and Tables

**Table 1:** Park areas and number of observation zones (prepared by the authors).

Park Name	Total Park Area * (ha)	Number of Observation Zones
St Marks Park	4.73	7
Pilrig Park	6.89	17
London Road Gardens	4.13	27
Victoria Park	6.16	17
Leith Links West	9.97 **	10
Lochend Park	8.40	21
Harrison Park West	3.99	5
Kingsknowe Park	6.04	9
Curriemuirend Park	4.42	24
Spylaw Park	3.65	20

* Park areas are based on park boundaries shown in the City of Edinburgh Council Atlas 70. Non-park areas (e.g., building, construction areas) were not included in the calculation. ** It was decided that Leith community craft area, within the Leith Links West, would be included in the observation study because of its close relation with the rest of the park (the total area including the Leith community craft area is 10.76 ha).

**Table 2:** Physical and social activity categories (prepared by the authors).

Physical Activity Categories	**Sedentary Activities**	**Low Level Activities**	**Medium Level Activities**	**High Level Activities**
	Lying Down Picnicking Sitting	Standing	Walking	Ball Games Cycling Exercise Jogging Playing in Playground Running and Playing
Social Categories	**Alone Activities**	**Couple Activities**	**Family Activities**	**Group Activities**

**Table 3:** Comparison of total park users observed in 2019 and 2020 by park and by observation category, the distribution of users in each category in each year, and statistical test results on the difference between the two years (data collected and table prepared by the authors).

Variables		Categories	2019	2020	Difference between Survey Waves
TOTAL			3025	5845	
Visited Park					
		St Marks Park	132 (4.4%)	284 (4.9%)	χ^2^(9) = 407.093 *p* < 0.05
		Pilrig Park	331 (10.9%)	832 (14.2%)	
		London Road Gardens	195 (6.4%)	236 (4.0%)	
		Victoria Park	613 (20.3%)	1444 (24.7%)	
		Leith Links West	901 (29.8%)	1005 (17.2%)	
		Lochend Park	201 (6.6%)	655 (11.2%)	
		Harrison Park West	396 (13.1%)	609 (10.4%)	
		Kingsknowe Park	15 (0.5%)	261 (4.5%)	
		Curriemuirend Park	28 (0.9%)	5 (0.1%)	
		Spylaw Park	213 (7.0%)	514 (8.8%)	
Session					
		Morning	567 (18.7%)	1183 (20.2%)	χ^2^(2) = 9.428 *p* < 0.05
		Afternoon	1268 (41.9%)	2256 (38.6%)	
		Evening	1190 (39.3%)	2406 (41.2%)	
Age					
		0–4	165 (5.5%)	360 (6.2%)	χ^2^(8) = 121.630 *p* < 0.05
		5–10	390 (12.9%)	1012 (17.3%)	
		10–15	210 (6.9%)	503 (8.6%)	
		16–24	376 (12.4%)	862 (14.7%)	
		25–34	833 (27.5%)	1464 (25.0%)	
		35–44	574 (19.0%)	1090 (18.6%)	
		45–54	288 (9.5%)	330 (5.6%)	
		55–64	114 (3.8%)	163 (2.8%)	
		65+	75 (2.5%)	61 (1.0%)	
Gender					
Overall					
		Female	1499 (49.6%)	2879 (49.3%)	χ^2^(1) = 0.071 *p* > 0.05
		Male	1526 (50.4%)	2966 (50.7%)	
Adults					
		Female	1171 (51.8%)	2191 (55.2%)	χ^2^(1) = 6.602 *p* < 0.05
		Male	1089 (48.2%)	1779 (44.8%)	
Activity					
		Lying Down	105 (3.5%)	119 (2.0%)	χ^2^(10) = 162.579 *p* < 0.05
		Picnicking	157 (5.3%)	235 (4.0%)	
		Sitting	633 (21.2%)	1629 (27.9%)	
		Standing	594 (19.9%)	1140 (19.5%)	
		Walking	983 (32.9%)	1388 (23.8%)	
		Ball Games	108 (3.6%)	309 (5.3%)	
		Cycling	64 (2.1%)	123 (2.1%)	
		Exercise	24 (0.8%)	108 (1.8%)	
		Jogging	36 (1.2%)	74 (1.3%)	
		Playing in Playground	208 (7.0%)	561 (9.6%)	
		Running and Playing	73 (2.4%)	156 (2.7%)	
Broad Activity					
		Sedentary Activities	895 (30.0%)	1983 (33.9%)	χ^2^(3) = 101.161 *p* < 0.05
		Low-Level Activities	594 (19.9%)	1140 (19.5%)	
		Medium-Level Activities	983 (32.9%)	1388 (23.8%)	
		High-Level Activities	513 (17.2%)	1331 (22.8%)	
Social					
		Alone	1025 (33.9%)	1135 (19.4%)	χ^2^(3) = 237.194 *p* < 0.05
		Couple	554 (18.3%)	1326 (22.7%)	
		Family	768 (25.4%)	1618 (27.7%)	
		Group	678 (22.4%)	1766 (30.2%)	
Presence of Dogs					
		Without Dogs	2564 (84.8%)	5263 (90.0%)	χ^2^(1) = 53.607 *p* < 0.05
		With Dogs	461 (15.2%)	582 (10.0%)	
Park Usage					
		Social	109 (3.6%)	452 (7.7%)	χ^2^(5) = 186.517 *p* < 0.05
		Playground	442 (14.6%)	959 (16.4%)	
		Sports and Fitness	79 (2.6%)	297 (5.1%)	
		Woodlands	25 (0.8%)	67 (1.1%)	
		Paths	992 (32.8%)	1270 (21.7%)	
		Other Park Areas	1378 (45.6%)	2800 (47.9%)	
Group Size					
			M = 3.17 Mdn = 2	M = 3.44 Mdn = 2	U = 7,398,991 z = −13.3, *p* < 0.05
Experienced Density of People					
	Without Any Group Members				
		2 m	M = 0.11 SE = 0.01	M = 0.08 SE = 0.006	t(5800.5) = 2.36 *p* < 0.05, r = 0.03 *
		5 m	M = 0.51 SE = 0.029	M = 0.63 SE = 0.029	t(8035.7) = −3.08 *p* < 0.05, r = 0.03 *
		10 m	M = 1.42 SE = 0.063	M = 2.19 SE = 0.07	t(8467.5) = −8.19 *p* < 0.05, r = 0.09 *
		20 m	M = 3.24 SE = 0.116	M = 6.61 SE = 0.152	t(8855.5) = −17.59 *p* < 0.05, r = 0.18 *
		50 m	M = 10.44 SE = 0.24	M = 22.82 SE = 0.331	t(8888.1) = −30.28 *p* < 0.05, r = 0.31 *
	With Any Group Members				
		2 m	M = 0.95 SE = 0.026	M = 1.03 SE = 0.022	t(7078.9) = −2.40 *p* < 0.05, r = 0.03 *
		5 m	M = 1.92 SE = 0.049	M = 2.4 SE = 0.044	t(7447.3) = −7.33 *p* < 0.05, r = 0.08 *
		10 m	M = 3.15 SE = 0.079	M = 4.24 SE = 0.081	t(8106.9) = −9.59 *p* < 0.05, r = 0.11 *
		20 m	M = 5.25 SE = 0.129	M = 8.87 SE = 0.158	t(8760.8) = −17.78 *p* < 0.05, r = 0.19 *
		50 m	M = 12.61 SE = 0.245	M = 25.25 SE = 0.335	t(8886.7) = −30.45 *p* < 0.05, r = 0.31 *

* Welch’s t-test was performed since the equality of variances was not met. Note 1: Park maintenance staff observed in parks were not included in the analysis; however, they were included in the calculation of the experienced density of people. Note 2: Individuals observed as ‘mobile’ and categorised as ‘other’ in the activity and broad activity categorisation were not included in the analysis of activity and broad activity categories.

## Data Availability

Data will be made available on request.
